# Gamma Radiation Image Noise Prediction Method Based on Statistical Analysis and Random Walk

**DOI:** 10.3390/s22197325

**Published:** 2022-09-27

**Authors:** Dongjie Li, Haipeng Deng, Gang Yao, Jicheng Jiang, Yubao Zhang

**Affiliations:** 1 Heilongjiang Provincial Key Laboratory of Complex Intelligent System and Integration, Harbin University of Science and Technology, Harbin 150080, China; 2 Key Laboratory of Advanced Manufacturing and Intelligent Technology, Ministry of Education, Harbin University of Science and Technology, Harbin 150080, China; 3 Heilongjiang Institute of Atomic Energy, Harbin 150086, China

**Keywords:** gamma radiation, Gaussian mixture model, random walk, image noise prediction

## Abstract

The gamma radiation environment is one of the harshest operating environments for image acquisition systems, and the captured images are heavily noisy. In this paper, we improve the multi-frame difference method for the characteristics of noise and add an edge detection algorithm to segment the noise region and extract the noise quantization information. A Gaussian mixture model of the gamma radiation noise is then established by performing a specific statistical analysis of the amplitude and quantity information of the noise. The established model is combined with the random walk algorithm to generate noise and achieve the prediction of image noise under different accumulated doses. Evaluated by objective similarity matching, there is no significant difference between the predicted image noise and the actual noise in subjective perception. The ratio of similarity-matched images in the sample from the predicted noise to the actual noise reaches 0.908. To further illustrate the spillover effect of this research, in the discussion session, we used the predicted image noise as the training set input to a deep residual network for denoising. The network model was able to achieve a good denoising effect. The results show that the prediction method proposed in this paper can accomplish the prediction of gamma radiation image noise, which is beneficial to the elimination of image noise in this environment.

## 1. Introduction

With the development of nuclear technology and the industrialization process in China, nuclear and radiation technology are widely used in aerospace, electric power, industrial agriculture, and other fields to create substantial economic benefits [1]. In the process of reactor repair and maintenance, daily supervision of cobalt source devices, and emergency disposal of radiation accidents, it is imperative to provide accurate visual information for operators. Image sensors are exposed to high energy in a gamma radiation environment, which will produce accumulated dose effects and transient ionization effects, causing abnormalities in the output of the image element [2], so that the resulting image will have serious noise. When particles in the gamma radiation environment are incident on the semiconductor device, the charged particles can excite some bound electrons from the valence band to the conduction band through the ionization process, resulting in many electron-hole pairs, forming a dense ionization trail. These electron-hole pairs generated by charged particle radiation have an impact on the performance of the semiconductor device, resulting in the generation of noise in the image. In this process, the secondary electrons are small in extent and low in energy, but they are also a cause of noise generation. When the secondary electrons replace the original electrons in the image element, this will cause transient pulse currents that will macroscopically manifest as bright spots on the image [3]. If the radiation causes lattice defects or displacement damage to the sensor, resulting in degradation of its function, the corresponding image element will work abnormally and produce temporary or permanent abnormal output in the subsequent image [4]. The ^60^Co source emits high-energy rays consisting of many gamma photons during the decay process. These gamma photons have strong penetrating properties and will transfer energy to surrounding atoms or electrons through the photoelectric effect, primary secondary Compton scattering, electron pair effect, and other processes in the process of penetrating the sensor, resulting in a high number of carriers in the image element penetrated by the gamma photons, which leads to a bright block noise in the scene image in the gamma radiation environment. The effects of gamma radiation on image sensors can be seen as three sequential processes: scattering of radiation particles, injection of particles into the sensor, and the output of an abnormal image signal from the sensor. Anti-radiation reinforcement methods can improve the tolerance of electronic components to radiation, but this will bring high costs and increase the size of the device structure [5].

The image quality directly affects the accuracy of the subsequent processing results. To achieve better processing results, we will enhance the image. Image denoising is an important part of image enhancement. It clarifies the image with contextual information [6]. The denoising method is determined by the noise characteristics. The classical denoising method has good generalization ability, but the denoising effect is average. With the development of artificial intelligence, algorithms based on deep learning are being increasingly applied to image denoising. Sun [7] proposed a radiation image noise reduction method based on convolutional neural networks to fit the mapping relationship between noisy images and pure images, which can retain the image details to the maximum extent while filtering out noise compared with traditional image noise reduction methods. Chen [8] estimates the noise distribution of noisy images and generates noise samples. Image denoising by training a deep convolutional neural network with noisy samples. 

The image denoising algorithm based on deep learning has great advantages in denoising effect and running time. Its denoising model requires clear images without noise and noisy images as positive and negative training samples for testing. However, it is difficult to conduct positive and negative sample image acquisition experiments for sudden situations, such as the movement of living bodies and cargo dumping, in the special environment of gamma radiation, which causes the scarcity of data samples under specific conditions. Traditional data enhancement methods for data expansion yield images with less variability and limited supervised information [9]. Common noises such as Gaussian noise, pretzel noise, gamma noise, impulse noise, and other common noises have artificial methods of generation, while the noise that appears in images in the gamma radiation environment is patchy [10] and differs from these noises in terms of morphology and amplitude.

The establishment of noise models is of great significance for denoising. For example, (1) the predicted image noise can be used as a training set for deep learning models for image denoising, and (2) the predicted image noise can be used to evaluate the effectiveness of denoising algorithms. Yuan [11] has established a noise model for sonar images through statistical analysis of sonar noise, which provides an effective aid for denoising. In the medical field, how to obtain clearer CT images at low dose and the effects of X-rays on the human body have been of great interest. Moser [12] gave a full explanation of the relationship between clarity and dose in CT images. Zhang [13] built an optimal exposure index for X-rays using clinical diagnosis as a guide. To improve the clarity of low-dose medical CT images, Qin [14] established a statistical model of noise, which was used to reduce the effect of noise on medical reconstruction images. The exploration of X-rays can promote the development of medical imaging. Then the exploration of gamma rays can promote the development of image denoising in industry. In the gamma radiation environment, the amplitude, amount, and shape of image noise in these gamma radiation environments are counted to provide a reference for image processing processes such as image denoising in gamma radiation environments to eliminate the effects of noise on images in gamma radiation environments.

In view of this situation, this paper improves the threshold selection method of the three inter-frame difference method and adds the Canny operator to detect the noise region, the amplitude and quantity information of the noise, statistical analysis, and the establishment of a Gaussian mixture model of gamma radiation noise. The established model is combined with the random walk algorithm to generate noise and finally predict the image noise under different accumulated doses. The results show that the method has a good prediction effect for gamma radiation image noise. The predicted image noise has a high similarity to the actual radiation image noise.

## 2. Noise Detection and Extraction

The image noise includes readout noise and intrinsic noise [15]. When the camera has good initial performance and a stable line connection, the intrinsic noise component is small. The image acquisition device is a branded CMOS camera with a resolution of 2560 × 1440, a frame rate of 25 fps, a minimum illumination of 0.005 Lux and an image element size of 2 µm, with a shutter range between 1/3 and 1/100,000 s.

Our team used the camera for the experimental acquisition of noise data in the II cobalt source device of the Heilongjiang Institute of Atomic Energy. The radiation dose rate at the experimental position was 6 Gy/h, the accumulated doses was 50 Gy, 100 Gy, 300 Gy, 500 Gy, and 1000 Gy. The distance between the image acquisition equipment and the center of the radiation source was 7 m, and the diameter of the radiation source was 40 cm. We selected 1600 images, each of the same accumulated doses, for a total of 8000 images.

The prerequisite for data analysis of noise in a gamma radiation environment is that the detected noise should be free of omission and complete. This meaning that the effective information should not be identified as noise. In the gamma radiation environment, the image noise has the characteristics of obvious jump and transients. The central area is usually a bright spot and the edge areas have a high contrast with the adjacent areas. The camera lens cover is closed during the image acquisition, so that there are no interfering objects in the field of view to avoid the noise in the actual image affecting the analysis results.

The three inter-frame difference method was improved by the authors of [16] by adding morphological transformations. When using this method for image noise retrieval in a gamma-radiation environment, due to the high dose of radiation, some noise will appear in the same location of the image in multiple consecutive frames. The three-frame differential algorithm will miss such noise and cause incomplete noise contour recognition and the occurrence of “hollow” or “double shadow” phenomenon [17]. Moreover, the inclusion of morphological transformations causes the edges of the noise to be blurred to ensure that the amount of noise is not missed and the contour is complete. In this paper, the three inter-frame difference method is improved and combined with the edge detection operator to perform noise location retrieval. The aim of the traditional inter-frame difference method is to acquire the dynamic target and filter out the noise, whereas in this case we aim to acquire the location of the noise, so the sum operation of the inter-frame difference is changed to an operation to extend the noise retrieval region. The thresholds for traditional inter-frame differencing are usually defined empirically, and a large or small threshold can result in incorrect retrieval of the target. This paper uses OSTU for adaptive selection of thresholds, which is calculated as follows:

(1) $$T=\max[w_{0}\times {\left(u_{0}-u\right)}^{2}+w_{1}\times {\left(u_{1}-u\right)}^{2}]$$

In this equation, $w_{0}$ is the proportion of background pixels in the image, $u_{0}$ is the average greyscale of the background pixels, $w_{1}$ is the proportion of foreground pixels in the image, $u_{1}$ is the average greyscale of the foreground pixels, and u is the average greyscale of the whole image.

The retrieval results of the improved three-frame difference method are fused with Canny, which can preserve the noise quantization information and the noise local neighborhood image.

**Figure 1:**
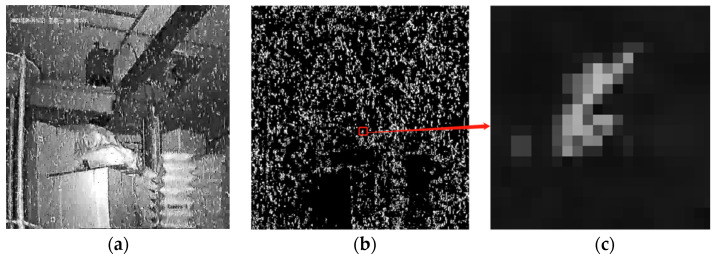
Detection and extraction of noise: (**a**) Images of radiation contamination; (**b**) Binarization image of noise position and (**c**) Individual noise contour.

**Figure 2:**
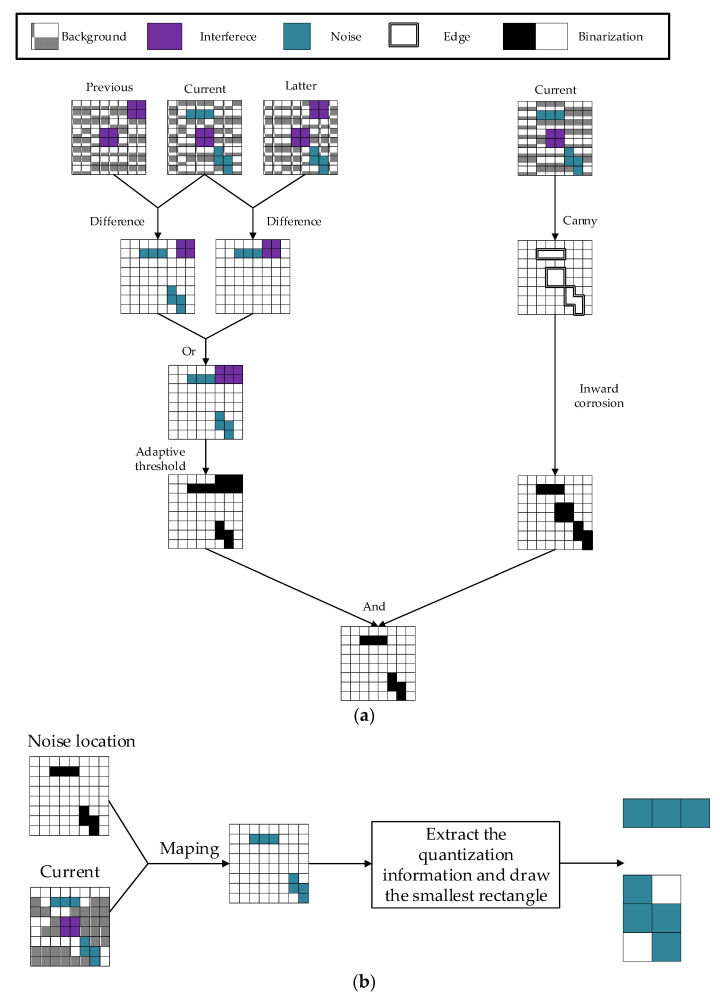
Image noise recognition and segmentation: (**a**) Detecting the location of noise; (**b**) Saving information about local noise.

The noise segmentation and quantization extraction are divided into two steps: (1) Detecting the location of noise. To take advantage of the outlier features of noise and the characteristics of small internal differences in noise, we use the inter-difference method, and the Canny detection algorithm in parallel operation to obtain the accurate position of noise in the current image. (2) Save local information of noise. The regional position of the noise is mapped with the current image. To obtain the local contour image of the current image noise, the local external minimum rectangle of the noise contour is drawn and saved in BMP format, where the quantization information is saved to a text file in the order of the noise arrangement. Figure 1 shows the results of the extraction of real image noise in a gamma-ray environment using the algorithm elaborated in Figure 2.

## 3. Modeling and Prediction of Radiation Image Noise

Radiation image noise modeling and prediction consists of two parts: (1) Constructing a radiated noise model, using various functions to fit the amplitude and quantity characteristics of the noise, and evaluating the fit effect using the coefficient of determination (R-square) and root mean square error (RMSE) tests. The RMSE is also called the standard deviation of the fit of the regression system, which is obtained by squaring the mean of the “sum of squares” of the fitted data and the original data, and the smaller the value is, the closer the fitted data is to the original data, and the better the model fit [18]. (2) Image noise generation method and prediction, based on the results of statistical analysis, the random walk algorithm was used to generate noise by setting parameters such as noise seed, number of walks, and range of walks, and to predict the image noise under different accumulated doses from 50 Gy to 1000 Gy.

### 3.1. Noise Amplitude Analysis

**Figure 3:**
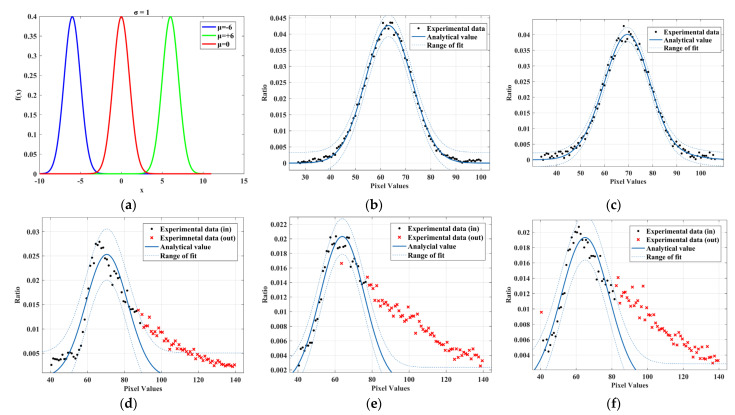
Noise amplitude fitting of a one-dimensional single Gaussian curve under different accumulated doses: (**a**) Gaussian curve; (**b**) 50 Gy; (**c**) 100 Gy; (**d**) 300 Gy; (**e**) 500 Gy and (**f**) 1000 Gy.

The noise amplitude distribution in images in common environments is commonly described by a single Gaussian model (SGM) [19], using a one-dimensional single Gaussian function to fit the amplitude in the extracted noise data. Figure 3 corresponds to an example curve of SGM and the fit curve to the noise amplitude using $f(x)=k\frac{1}{\sqrt{2\pi }\sigma }e^{-{\left(x-u\right)}^{2}/2\sigma^{2}}$ for fitting curves to noise amplitudes at different accumulated doses, where μ determines the location and σ determines the amplitude [20], and the resulting fitted covariates are shown in Table 1.

The distribution of noise quantities in amplitude at different accumulated doses is given in Figure 3. At an accumulated dose below 300 Gy, the image noise amplitude is concentrated between 40 and 90, fluctuating around the mean value. The fit result is good as seen from the R-square and RMSE metrics. The effects of gamma radiation on image sensors can be attributed to radiation-induced transient ionization effects and accumulated dose effects in image sensors [21]. The transient ionization effect leads to an increase in charge at the sensor potential, which produces bright spots in the image. The accumulated dose effect causes a drift in the flat band voltage and threshold voltage, changes the conductivity of the semiconductor, and increases the drain current [22], resulting in temporary or long-term abnormal output of some elements. When the accumulated doses reach 300 Gy, the noise amplitude begins to be biased towards higher areas. As the accumulated dose continues to increase, the influx of the accumulated dose effect becomes prominent and the image sensor begins to experience abnormalities in the operation of some image elements, with the effect of intrinsic mode noise gradually increasing. The overall distribution curve of the noise amplitude has an asymmetrical character, skewing towards the higher amplitude regions. The SGM can no longer describe the amplitude distribution of the noise.

**Figure 4:**
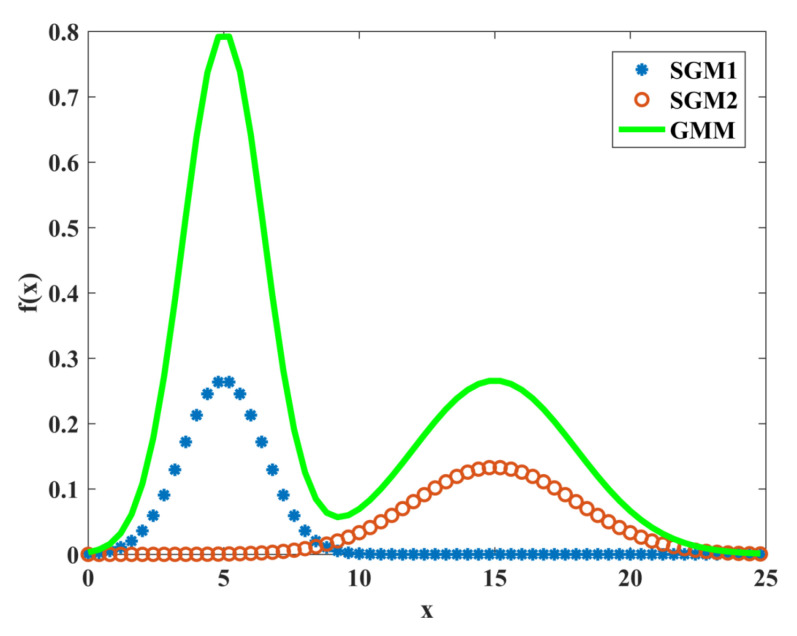
Two-component one-dimensional mixed Gaussian curve.

**Figure 5:**
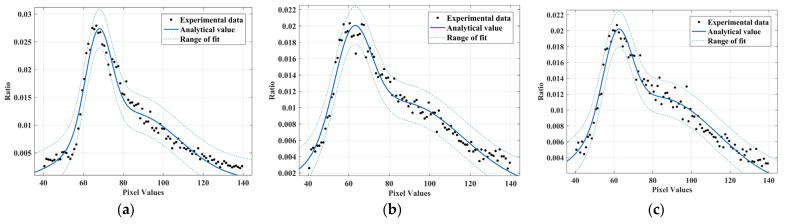
Fitting of a two-component one-dimensional mixture Gaussian curve to noise amplitude under high accumulated doses: (**a**) 300 Gy; (**b**) 500 Gy and (**c**) 1000 Gy.

A Gaussian mixture model (GMM) can be obtained by the linear superposition of several single Gaussian models with data conforming to a mixture Gaussian distribution [23]. An example curve of a two-component one-dimensional mixed Gaussian model is given in Figure 4. SGM1 and SGM2 are one-dimensional single Gaussian distribution curves with a mean of 5 and a variance of 1.5 and a mean of 15 and a variance of 3, respectively, and the outgoing one-dimensional mixed Gaussian distribution model GMM with a linear superposition of SGM1 and SGM2 with weight coefficients of 2 and 3 are used at higher accumulated doses using a two-component one-dimensional mixed Gaussian model. The noise amplitude is fitted. The fitting function is shown in Equation (2) and $k_{1}$ and $k_{2}$ denote the weight of each component. Figure 5 corresponds to the fitting curve of the noise amplitude at high accumulated doses using the two-component one-dimensional hybrid Gaussian function, and the resulting fitted parameters are shown in Table 2.

(2) $$f(x)=k_{1}\frac{1}{\sqrt{2\pi }\sigma_{1}}e^{-{\left(x-u_{1}\right)}^{2}/2\sigma_{1}{}^{2}}+k_{2}\frac{1}{\sqrt{2\pi }\sigma_{2}}e^{-{\left(x-u_{2}\right)}^{2}/2\sigma_{2}{}^{2}}$$

According to Figure 5 and Table 2, the fitting of the image noise amplitude at high accumulated doses using a two-component mixture Gaussian distribution is significantly better than that of a one-dimensional single Gaussian distribution. The experimental data are all within the critical range of the fit. This demonstrates that the two-component mixture Gaussian model can fit the image noise amplitude at high accumulated doses.

### 3.2. Noise Quantity Analysis

**Figure 6:**
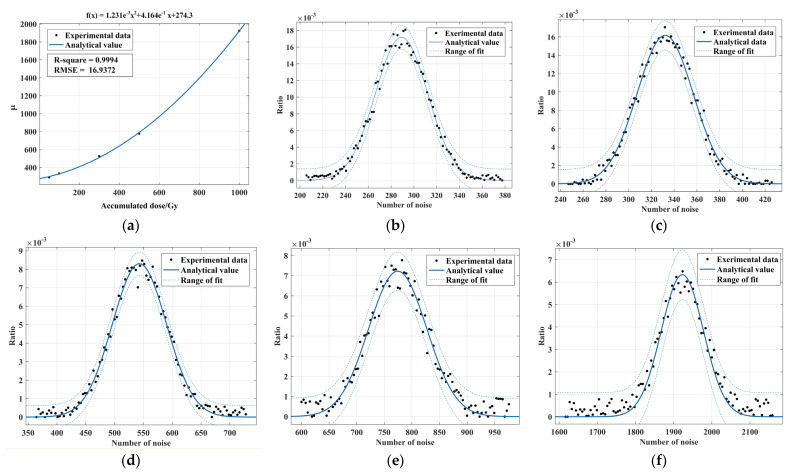
The number of noise distributions for (**a**) Different accumulated doses; (**b**) 50 Gy; (**c**) 100 Gy; (**d**) 300 Gy; (**e**) 500 Gy and (**f**) 1000 Gy.

From separate data analysis of the range of image noise at different accumulated doses and the distribution of the number of noises in consecutive image frames at the same accumulated doses. It can be concluded that the number of noisy images increases non-linearly with the increase of the accumulated doses. Figure 6 shows the trends and fitted curves for the mean value of noise quantities at different accumulated doses. As shown in Figure 6b–f, under the same accumulated doses, the number of radiation image noise shows a strong regularity. The number of noise fluctuates around the mean value. The curve shows the characteristics of the spike large slope shake, with a strong symmetry. The fitting parameters are shown in Table 3. According to the fitting effects in Figure 6b–f, as well as the R-square and RMSE in Table 3, it can be concluded that the one-dimensional single Gaussian model can achieve better noise fitting under the same accumulated doses. 

### 3.3. Prediction of Noise

The prediction of gamma radiation image noise is based on the quantity, amplitude, and shape characteristics of the noise. By statistical analysis of the noise and image processing, gamma radiation image noise has the characteristic of appearing at random locations. For the same accumulated doses, the individual noise connected areas are of ap-proximate size and irregular shape, and the amplitude intensity follows a one-dimensional two-component Gaussian mixture distribution. The number of image frames possessing noise quantity is consistent with the one-dimensional single Gaussian distribution. The amplitude and quantity of noise are predicted based on the statistical analysis results. The noise shape is predicted by the random walk algorithm.

The application of the random walk algorithm to images is based on graph theory [24], in which the structure of the graph, in which any two nodes may be related, consists of a set K of nodes and a set E representing the relationship of adjacent nodes, denoted as $G=\left(V,E\right)$. The image is considered as a pure discrete matrix, which can be represented as a graph with image dimensions w×h nodes and (w−1)×(h−1) edges, and each edge is set with a certain number of weights, indicating the probability of the node traveling to that edge, where the formula for finding the walk weights by the Gaussian function is as follows:

(3) $$w_{ij}=\exp(-{\frac{\left\|p_{i}-p_{j}\right\|}{2a^{2}}}^{2})$$

$p_{i}$ and $p_{j}$ are the magnitudes of pixel x and neighbor x+1, respectively, a is a custom parameter, and a weight of 0 means that the random walk will not proceed along this edge walk. The random walk on the image is essentially a Dirichlet problem to find whether the boundary is 0 or 1.

In the prediction process, each block noise can be regarded as composed of several different numbers of noise units and has a noise seed. The noise seed is the starting point of the block noise formation process, to carry out the noise generation under different accumulated doses. The position of the noise seeds is random *x*, *y*, and the number of noise seeds is *N*:

(4) N(Gy)=Choice(Np(Gy),Nnmn(Gy))

The formula represents the number of noises Nnmn selected with probability Np, Gy represents the accumulated dose:

(5) $$Np=k\frac{1}{\sqrt{2\pi }\sigma }e^{-{\left(x-u\right)}^{2}/2\sigma^{2}}$$

The parameters in the above equation are determined according to Table 3.

The amplitude of noise seeds is *F*:

(6) F(Gy)=Choice(Fp(Gy),Famp(Gy))

The formula represents the amplitude of noise Famp selected with probability Fp, Gy represents the accumulated dose:

(7) $$Fp=k_{1}\frac{1}{\sqrt{2\pi }\sigma_{1}}e^{-{\left(x-u_{1}\right)}^{2}/2\sigma_{1}{}^{2}}+k_{2}\frac{1}{\sqrt{2\pi }\sigma_{2}}e^{-{\left(x-u_{2}\right)}^{2}/2\sigma_{2}{}^{2}}(if(Gy<=100),k_{2}=0)$$

The parameters in the above equation are determined according to Table 1 and Table 2.

**Figure 7:**
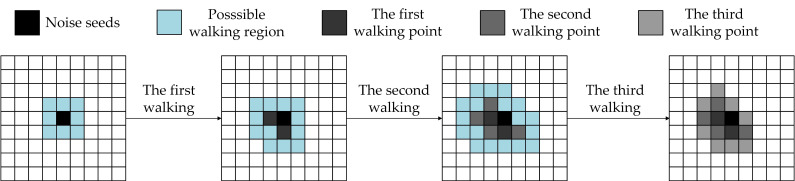
Random walk process of isolated noise seeds.

The noise seeds in the range of image size m × n, through the random walk towards the noise seed neighborhood diffusion. Due to the random nature of the noise shape, the noise seeds are spread across the image using random boundary weights and finally produce the same size as the image as the to be processed image containing radiation noise. Figure 7 shows the random walking process of isolated noise seeds.

The pseudo code for the noise prediction is shown below (see Algorithm 1):
**Algorithm 1:** Gamma radiation image noise prediction**Input:** Pure image M;    Radiation accumulated dose Gy; **Output:** Noise image M* 1: S←ChoiceNum(Np(Gy),Nnum(Gy)); 2: F←ChoiceAmp(Fp(Gy),Famp(Gy)); 3: X,Y←RandomChoice(M.rows,M.cols,S); 4: k←0; 5: l←0; 6: **while** (k < S) **do** 7:   W←Random(0,D[k]) 8:   Rw←RandomWalk(X[k],Y[k],W) 9:   **while** (l < W) **do** 10:     **if** Rw.X[l] < M.rows **and** Rw.Y[l] < M.cols then 11:       M[Rw.X[l]][Rw.Y[l]]←F[k] 12:     **else** 13:       Continue 14:     **end if** 15:     l←l + 1 16:  **end while** 17:  k←k + 1 18: **end while** 19: M*←M20: **return** M*

## 4. Results

To verify the prediction effect of this paper on the image noise of the gamma radiation environment, we have conducted both subjective and objective evaluations of the effectiveness of noise prediction.

**Figure 8:**
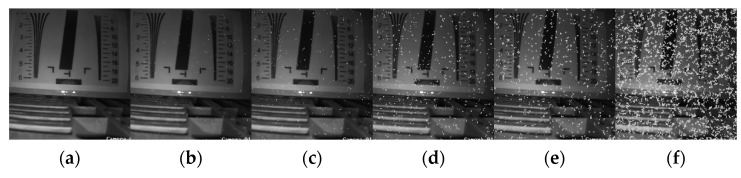
Prediction of irradiation chamber image noise under different accumulated doses: (**a**) Original image; (**b**) 50 Gy; (**c**) 100 Gy; (**d**) 300 Gy; (**e**) 500 Gy and (**f**) 1000 Gy.

**Figure 9:**
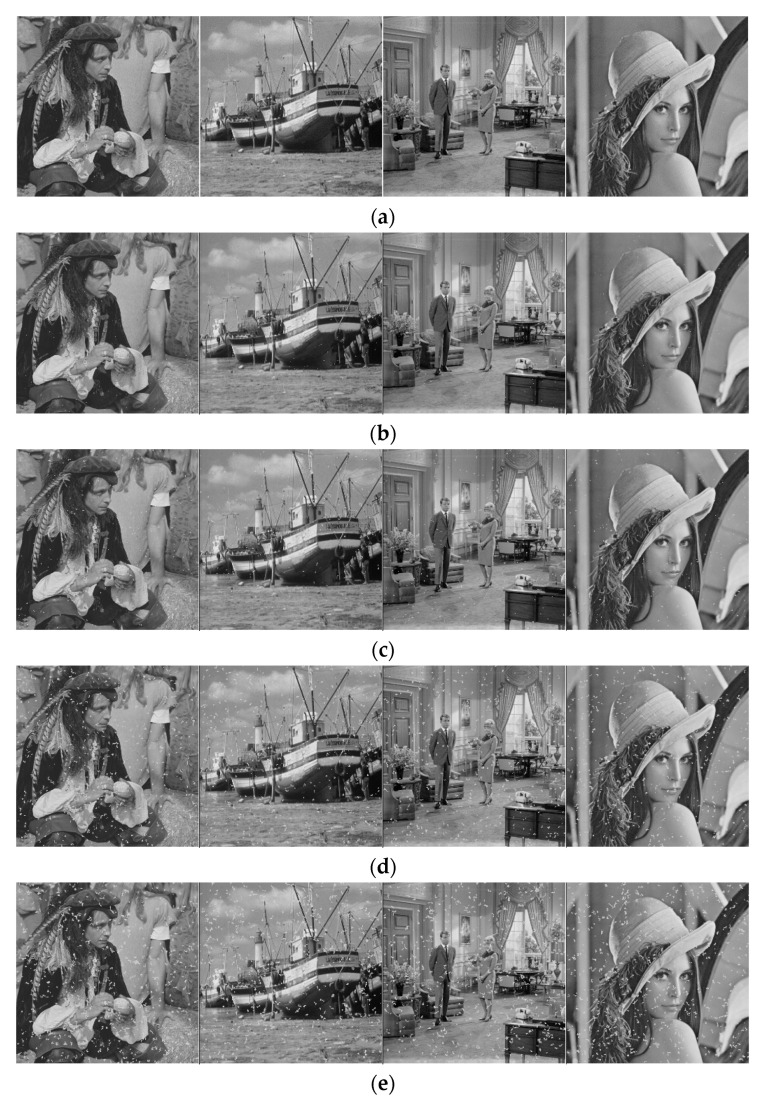

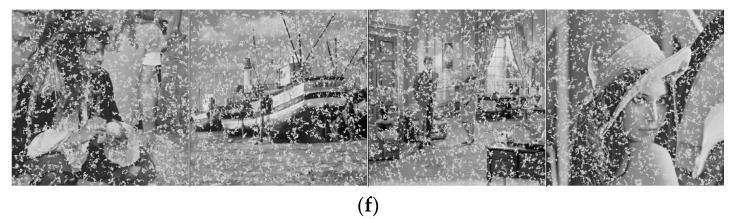
Prediction of natural image noise under different accumulated doses (original images from the Set 12 [25] dataset): (**a**) Original image; (**b**) 50 Gy; (**c**) 100 Gy; (**d**) 300 Gy; (**e**) 500 Gy and (**f**) 1000 Gy.

The noise prediction of irradiated room images and natural images at different accumulated doses using this method is given in Figure 8 and Figure 9.

With the predicted images at different evaluation doses, it can be concluded that the predicted noise patches have better results. Subjective evaluation methods rely heavily on the subjective awareness of the evaluator, and the similarities and differences between the images cannot be quantified well. Objective methods of image quality evaluation are based on mathematical models that analyze the degree of difference and similarity based on reference images [26]. The objective evaluation method for evaluating the quality of predicted noise in this paper is to evaluate the probability of obtaining the actual radiated noise image from the actual radiated noise set, the predicted radiated noise set, and the Gaussian noise set by calculating the similarity. The specific steps are as follows:

1 Five hundred randomly selected as samples from the actual radiation noise set, 1000 selected as matching target dataset I $X_{500}$, 1000 randomly selected as matching target dataset II $O_{1000}$ from the predicted data, and 1000 randomly selected as matching target dataset III $G_{1000}$ from the Gaussian generated noise data;
2 Select one image from $X_{500}$ in turn and match the similarity in $O_{1000}$, $P_{1000}$, and $G_{1000}$. If the image with the highest similarity to the sample is from the target dataset $O_{1000}$ then it is denoted as m=−1, if the image with the highest similarity to the sample is from the target dataset $P_{1000}$ then it is denoted as m=1, if the image with the highest similarity to the sample is from the target dataset $G_{1000}$ then it is denoted as m=−0.2;
3 Repeat step two until all of $X_{500}$ has been traversed;
4 Sum $n=\sum_{x=1}^{200}m_{x}$ overall m and calculate the proportion $p=\frac{i}{j}$ of samples from $P_{1000}$ and $O_{1000}$;
5 Conduct different accumulated doses experiments on $X_{500}$, $O_{1000}$, $P_{1000}$, and $G_{1000}$.

The specific results are shown in Table 4

As can be seen from the table, under the accumulated dose of 300 Gy and below, some of the actual radiation noise similarity is close to Gaussian noise. When the accumulated dose becomes large, the noise is rarely or never discerned as Gaussian noise. Meanwhile, the predicted radiation noise in this paper is closer to the actual noise matching results. Under 1000 Gy, 262 samples were matched to the actual radiation noise dataset I, 238 samples with the predicted radiated noise were matched to the actual radiated noise dataset I, and 238 samples were matched to the predicted radiated noise sample set II. Both subjective and objective evaluation methods prove the effectiveness of the algorithm for gamma-radiation noise image prediction.

## 5. Discussion

With the development of unmanned machine systems in recent years, increased operations in harsh environments are being replaced by robots. The camera plays a very important role as the eyes of the robot. The noise in the image affects the normal operation of the robot. The predicted noise of an image is of importance as it can be used. For example, (1) the predicted image noise can be used as a training set for deep learning models for image denoising, and (2) the predicted image noise can be used to evaluate the effectiveness of denoising algorithms.

**Figure 10:**
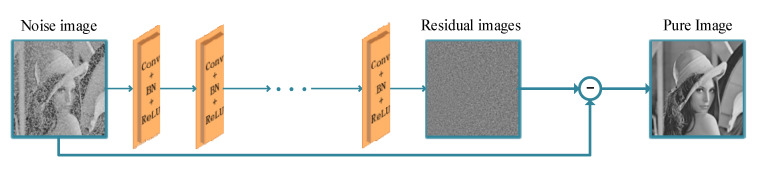
Structure of a deep residual network.

To further demonstrate the spillover effect of this paper’s research, we used a deep residual network denoising model from the literature [8]. The network structure is shown in Figure 10.

**Figure 11:**
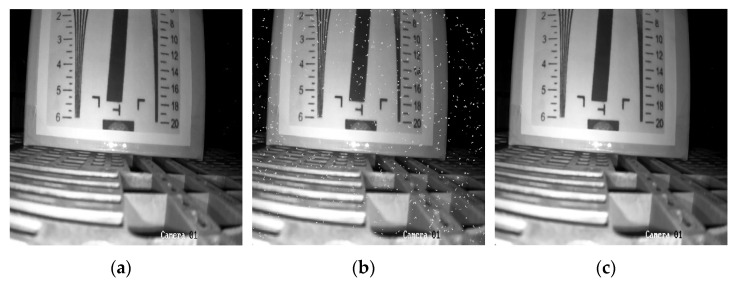
Denoising visualization (**a**) Original image; (**b**) Noise image; and (**c**) Denoised image (Deep Residual Network [8]).

After the predicted noisy images are fed into the network as a dataset for training, the real noisy images are used in the tests. The PSNR and SSIM were able to reach 35.472 and 0.971 respectively. The denoised visual results are shown in Figure 11.

The images generated using the prediction method in this paper were put into a deep residual network as the training set, which was able to achieve good denoising results. This result demonstrates some of the spillover implications of this paper’s research.

The experiments in this article provide some enlightenment for our future work. We start from the formation causes and nature of image noise in gamma radiation environments, to study the noise in gamma radiation images and provide new ideas for image denoise and robot operation in gamma radiation environments. Our method proposed in this paper predicts and evaluates the image noise at different accumulated doses in the gamma radiation environment. Although the method has been implemented in a single environment and controlled situation, the work is significant because it provides the results of radiation noise prediction in the environment of high accumulated doses. In the future, the unconventional image noise generated in other malignant environments should also be studied.

## 6. Conclusions

In order to complete the prediction of image noise in the harsh environment of a gamma radiation environment, this paper improves the threshold selection method of the three inter-frame difference method and introduces the Canny operator to detect the noise of the image to obtain the amplitude, location, and quantity information of the noise. Then we use probability statistics to process the amplitude and quantity information of the noise, build a statistical model of the noise, and combine the model with a random walk to generate the noise. Finally, we add the generated noise to the image and calculate the probability by similarity matching to objectively evaluate the noise prediction effect. In the discussion session, we used the predicted noisy image as a sample input to a deep residual network for training, and the network was able to achieve better denoising results. Some spillover effects of the research have been demonstrated. The results show that the predicted gamma radiation image noise in this paper can replace the real gamma radiation image noise in some scenarios, solving the characteristics that image noise in the gamma radiation environment is highly random and difficult to determine. It provides conditions for objective evaluation of the performance verification of the speckle noise denoising algorithm in the gamma radiation environment, which is conducive to the elimination of image noise in this environment and promotes the development of image processing in this harsh environment of gamma radiation.

## Figures and Tables

**Table 1:** Parameters of a one-dimensional single Gaussian fitting noise amplitude distribution.

Accumulated Dose (Gy)	*k*	*μ*	*σ*	R-Square	RMSE
50	1.000	63.19	9.24	0.9813	7.089 × 10^−4^
100	1.000	69.44	9.76	0.9727	1.152 × 10^−3^
300	0.6612	68.92	10.16	0.8899	1.161 × 10^−3^
500	0.6052	64.98	12.09	0.8796	1.427 × 10^−3^
1000	0.5937	65.26	13.42	0.8422	2.723 × 10^−3^

**Table 2:** Two-component one-dimensional Gaussian mixture fitting noise amplitude distribution parameters.

Accumulated Dose (Gy)	*k*_1_	*μ*_1_	*σ*_1_	*k*_2_	*μ*_2_	*σ*_2_	R-Square	RMSE
300	0.2887	67.48	6.47	0.7116	84.14	23.75	0.9522	1.066 × 10^−3^
500	0.2540	62.91	9.07	0.7462	86.82	29.47	0.9566	1.101 × 10^−3^
1000	0.2071	63.59	10.43	0.8013	82.92	30.62	0.9534	1.236 × 10^−3^

**Table 3:** One-dimensional single Gaussian fitting noise number distribution parameters.

Accumulated Dose (Gy)	k	μ	σ	R-Square	RMSE
50	1.000	289.2	22.3728	0.9869	4.092 × 10^−4^
100	1.000	332.5	24.5971	0.9879	4.256 × 10^−4^
300	1.000	546.7	46.9324	0.9765	5.328 × 10^−4^
500	1.000	776.5	50.8409	0.9649	5.226 × 10^−4^
1000	1.000	1923.8	57.2038	0.9592	5.864 × 10^−4^

**Table 4:** Matching results of similarity.

Accumulated Dose (Gy)	m = −1	m = 1	m = −0.2	n	*p*
50	227	182	91	−63.2	0.802
100	239	191	70	−62	0.799
300	251	211	38	−47.6	0.840
500	264	234	2	−30.4	0.886
1000	262	238	0	−24	0.908

## Data Availability

Not applicable.
